# Coverage of Gingival Fenestration Using Modified Pouch and Tunnel Technique: A Novel Approach

**DOI:** 10.1155/2013/902585

**Published:** 2013-07-07

**Authors:** Sunil Pendor, Vidya Baliga, A. Muthukumaraswamy, Prasad V. Dhadse, Kiran Kumar Ganji, Kaustubh Thakare

**Affiliations:** ^1^Department of Periodontics, Sharad Pawar Dental College and Hospital, Sawangi (M), Wardha, Maharashtra 442004, India; ^2^Department of Periodontics, Tamilnadu Government Dental College and Hospital, Chennai, Tamilnadu 600003, India

## Abstract

Gingival fenestration defects are a rare phenomenon. Gingival fenestration means the exposure of the tooth due to loss of the overlying bone and gingiva. Though treatment of mucosal fenestration occurring in association with chronic periapical inflammation has been reported previously, the occurrence and treatment of gingival fenestration have not been documented in great detail. This report describes the occurrence of a gingival fenestration that developed secondarily to a gutka chewing habit. Treatment of the fenestration along with coverage of an adjacent recession defect in a single-step procedure using a pouch and tunnel technique is described.

## 1. Introduction

Gingival fenestrations are of uncertain etiology and have rarely been reported in the dental literature [1]. Gingival fenestration defects may create problems regarding plaque control, root hypersensitivity, and esthetics.

Gingival recession may be caused by periodontal disease, improper oral hygiene, frenal pull, bone dehiscence, improper restorations, tooth malposition, viral infections of the gingiva, and oral habits [2]. Recession defects are treated to resolve a variety of patient-centered concerns including, but not limited to, root sensitivity, increased potential for root caries, difficulty in plaque control, and esthetics [3]. Furthermore, it must be remembered that exposed roots are prone to abrasion and erosion.

Several innovations, modifications, and variations have been developed for surgical root coverage since Grupe and Warren [4] first described the laterally positioned flap. However, greater predictability of results became achievable only with the introduction of bilaminar connective tissue grafting techniques [5]. Raetzke in 1985 [6] described a bilaminar technique for isolated recession defects, creating an envelope or pouch around the recession area to receive connective tissue graft. Zabalegui and others [7] treated multiple gingival recessions by creating a tunnel under the areas of gingival recession to receive the connective tissue graft thus avoiding dissecting the intermediate papilla and improving blood supply to the flap. Highly successful root coverage was reported with these two techniques [6, 7]. 

Gutka is chiefly a mixture of powdered tobacco, areca nut (fruit of *Areca catechu*), and slaked lime (aqueous calcium hydroxide), usage of which is seen mainly in the Indian subcontinent and also enjoyed by immigrant communities settled in Europe and the United States [8]. Habitual gutka use has been associated with the occurrence of several oral mucosal disorders, including oral submucous fibrosis (OSF), oral cancer, and periodontal disease [9, 10]. 

The following case describes a rare situation of occurrence of a gingival fenestration in a gutka chewer. Treatment of the fenestration, as well as simultaneous root coverage of a class I recession defect on the adjacent tooth, creating a pouch and subjacent tunnel is described. A conservative incision technique for the preparation of the tunnel was used.

## 2. Case Report

**Figure 1:**
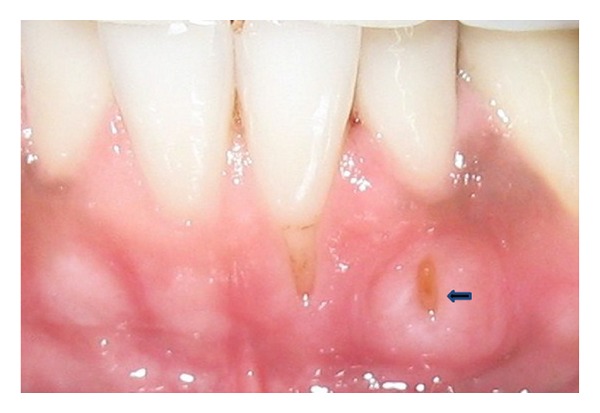
Intraoral photograph showing Miller's class I recession in the mandibular left central incisor and the fenestration defect (arrow).

A 27-year-old male reported to the authors' department with a complaint of hypersensitivity and burning sensation in the lower left front region since one month. History of two years of gutka chewing with placement of the gutka quid in the lower labial vestibule was reported. On examination, a portion of the root of mandibular left lateral incisor was visible through a gingival and alveolar bone fenestration on the labial surface (Figure 1). The fenestration was oval in shape, measuring approximately 4 × 2 mm in diameter. The area was free of pus and calculus, and the surrounding gingiva appeared whitish in color. All probing depth measurements around the tooth were within the normal limit, and the fenestrated area could not be probed through the gingival sulcus. The adjacent central incisor showed the presence of a Miller's class I recession [11] (Figure 1).

The patient was counselled regarding the harmful effects of gutka chewing and was referred to a tobacco-cessation center. Professional prophylaxis was performed 1 month after cessation of the habit. Informed consent was obtained from the patient.

**Figure 2:**
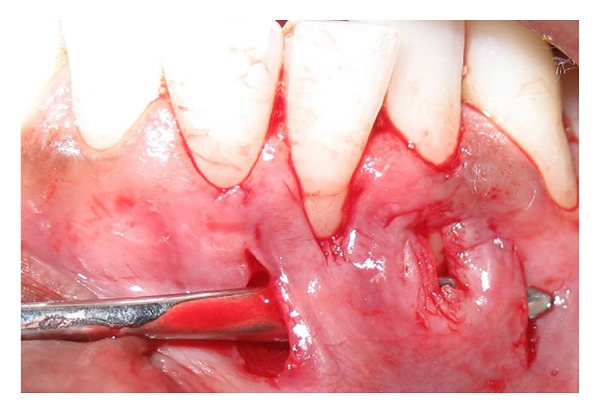
Partial thickness tunnel connecting two vertical incisions for fenestration coverage.

The surgery was initiated after administration of a local anesthetic agent (Xicaine, ICPA, Mumbai, India). A sulcular incision was given with a number 15C blade and a supraperiosteal pouch was created apically and laterally to the recession extending 3 to 5 mm in all directions. Two conservative submarginal vertical releasing incisions extending beyond the mucogingival junction were made on the mesial line angle of the right central incisor and distal line angle of the lateral incisor. A partial thickness tunnel was prepared extending horizontally, connecting the two vertical incisions (Figure 2). The tunnel was connected to the pouch, and the apical extension was carried beyond the mucogingival junction to facilitate the placement of the connective tissue graft.

**Figure 3:**
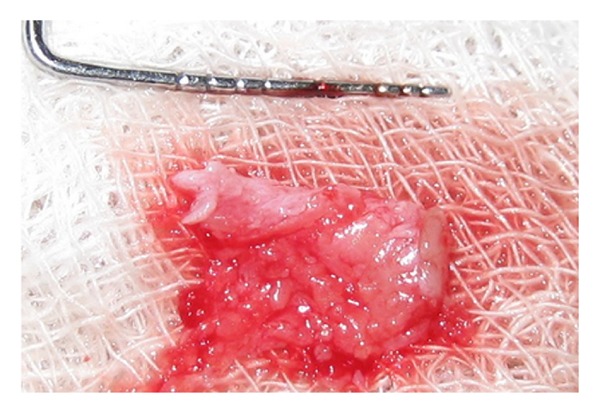
Harvested connective tissue graft.

Connective tissue graft of adequate dimensions (Figure 3) as measured with a template was procured from the palate (site: mesial aspect of the maxillary left first premolar to the distal aspect of the first molar) by the “trap door” approach [12] and the palatal donor site was sutured using a 4-0 nonresorbable, silk suture.

**Figure 4:**
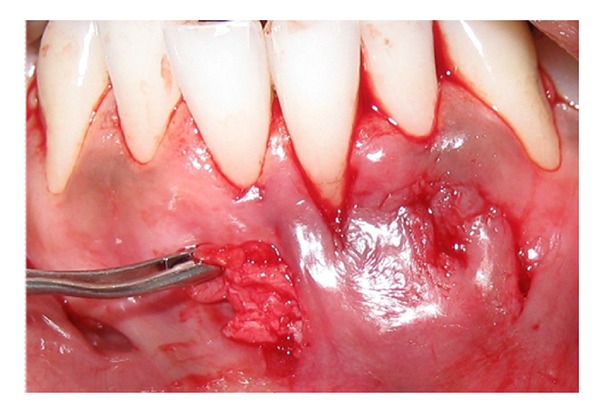
Placement of the connective tissue graft in the prepared tunnel.

**Figure 5:**
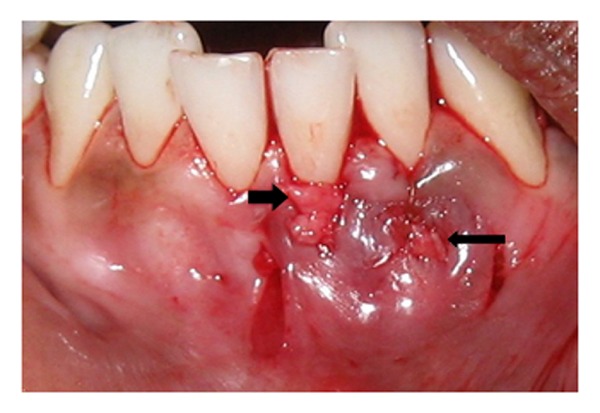
Extending the graft coronally into the prepared pouch (short and thick arrow-shows the graft placed in the prepared pouch); note coverage of the fenestration defect (long arrow) also.

**Figure 6:**
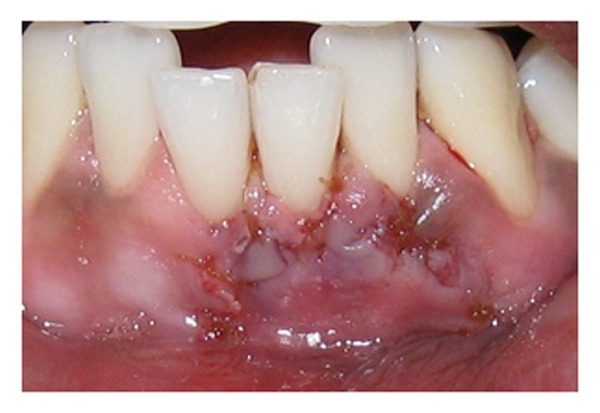
The graft was sutured by independent sling sutures and secured to the adjacent papillae by interrupted sutures in the recession area. Graft covering the fenestration defect was secured to the adjacent flap with interrupted sutures.

The harvested connective tissue graft was positioned in the prepared pouch by sliding it through the vertical incision area (Figure 4) and securing it to the adjacent interdental papillae using 5-0 absorbable suture (Vicryl, Ethicon, Johnson and Johnson, USA) and independent sling sutures. With the help of elevators the graft was further slid through the tunnel to cover the fenestration (Figure 5) and securing it to the adjacent flap using 5-0 Vicryl sutures. The vertical incisions were closed with interrupted sutures (Figure 6).

The patient was discharged after placement of a periodontal dressing (PerioCare, Pulpdent Corporation, Watertown, MA, USA). He was advised to refrain from mechanical cleansing of the surgical site, which could disturb initial healing, and instructed to rinse with 0.2% chlorhexidine gluconate solution (Hexidine, ICPA Health Care Products Ltd., Mumbai, India) twice a day for one minute. An analgesic was prescribed for the relief from any postsurgical pain.

**Figure 7:**
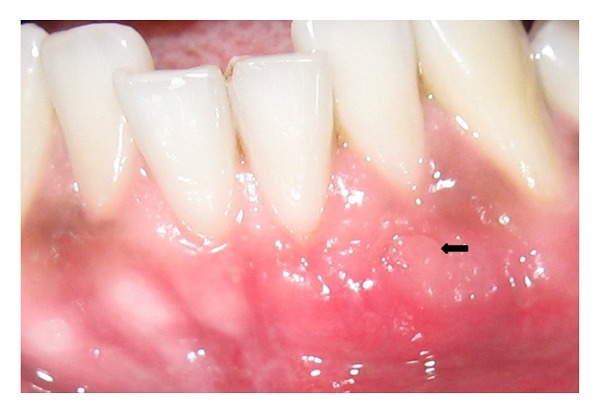
At 6-month followup the fenestration was completely covered and adequate root coverage of the recessed tooth was achieved.

Healing was uneventful. The sutures placed in the palate were removed after 1 week. The sutures placed on the buccal aspect and the periodontal pack was removed after 15 days. At the 1-month, 2-month, 3-month, and 6-month (Figure 7) postsurgical appointments, progressive adaptation of the edges of the graft to the surrounding tissues and increased morphologic and chromatic mimicking were observed. Six months after surgery, sulcular probing depth was less than 2 mm at the recession site, and no bleeding on probing was present. The gingival margin at the centre of labial surface was 0.5 mm short of the CEJ, but a recession depth reduction of 2.5 mm was seen (Prior to the surgery recession depth was 3 mm; after 6 months the residual recession at the site was 0.5 mm). The position of the mucogingival junction remained the same, but the amount of keratinized gingival on the left central incisor increased by 2.5 mm (1.0 mm before surgery, 3.5 mm after surgery). Though an indentation was seen at the fenestration area, complete coverage of the defect was completely seen. The patient was placed in maintenance program consisting of prophylaxis and motivation.

## 3. Discussion

Bilateral fenestration of the labial gingival tissue of the permanent mandibular central incisors has been reported in a developing child, which resulted in an apical positioning of the gingival margin even after maintenance of good oral hygiene over a 2-year period [2]. No etiologic factors were identified other than labial positioning of the teeth and the changes in gingival contour were noted as a part of the continuous process of remodeling.

Isolated gingival fenestration has also been reported to occur in association with cervical enamel projections (CEPs) [1]. The attachment between epithelium and enamel as a junction has been described to be composed of hemidesmosomes and a basal lamina [18, 19] thought to constitute an area of lessened resistance to plaque-associated inflammatory degradation [20]. 

Mucosal fenestrations have been reported previously [16, 17], and the probable etiological factors reported were extreme buccal inclination of root tips with very thin or nonexistent buccal cortical plate combining with chronic periapical inflammation [16, 17]. The various fenestration defects and the treatment modalities employed are summarized in Table 1.

Gutka contains fine grains of areca nut, which, besides causing mechanical injury to oral tissues, also allows ground tobacco to adhere to the traumatized mucosa, leading to morphologic changes and membrane damage [21]. In the present case the tooth with fenestration defect was free of periapical inflammation as indicated by radiographic and clinical examination. Oral hygiene performance was found to be adequate. Hence, it can be said that mechanical injury caused by gutka chewing may have led to the development of gingival fenestration. In addition, exposure of the tooth favored further deposition of plaque as well as subjecting it to constant mechanical injury caused by continued gutka chewing that prevented reformation of the mucosal covering. Mechanical injury combined with a labially aligned tooth may be the probable etiologic factors for occurrence of recession. 

Cessation of the gutka chewing habit may help to reduce the severity of the condition and may also prevent its progression; however, the defect caused required surgical intervention. Due to the presence of two adjacent defects, which would otherwise require two separate procedures for root coverage, were treated using a single-step procedure thus reducing patient morbidity.

One of the prerequisites for complete recuperation of the periodontal tissues is the maintenance of adequate vascularization in the flaps and grafts, which is an advantage offered by bilaminar techniques. Also, the presence of releasing incisions interrupts the superficial and intramural vascularization [5]. In the case discussed since the fenestration was located submarginally, the coronoapical extension of the releasing incisions was kept to the minimum necessary to facilitate access without involving the gingival margin and the papilla thereby improving the blood supply to the graft and further reducing patient morbidity.

Complete root coverage has been clinically defined on the basis of the following criteria [5]: (1) the marginal tissue reaches the level of the cementoenamel junction (CEJ); (2) clinical attachment is present; (3) sulcus depth is 2 mm or less; and (4) bleeding on probing is absent. Though primary coverage could not be attained in a small area, midlabially, one cannot rule out the possibility of secondary coverage that occurs by creeping attachment [22]. 

## 4. Conclusion

This case report shows the advantages in terms of predictability of coverage and esthetics when care is taken to ensure proper access through minimal releasing incisions. We believe that this technique is valuable when a single-step procedure is required to cover adjacent recession as well as a gingival fenestration defect. Given the widespread incidence of marginal tissue recession and associated esthetic concern of patients, the single-stage pouch and tunnel surgical technique may be beneficial in meeting the esthetic and functional demands of patients and also contribute to increased treatment acceptance and overall patient satisfaction.

## Figures and Tables

**Table 1:** Reported cases with fenestration and various treatment modalities employed.

Fenestration type	Region	Etiologic factors	Treatment
Gingival [2]	Mandibular centrals	Labial placement was considered part of development process	Observation—2-year period
Gingival [1]	Maxillary central incisor	Cervical enamel projections	Flap elevation with odontoplasty
Mucosal [13]	Maxillary central incisor	Chronic periapical inflammation	Re-root planing + chlorhexidine mouth rinsing
Mucosal [14]	Maxillary first molar	Chronic periapical inflammation, buccally inclined root tip	Full thickness mucoperiosteal flap with primary closure
Mucosal [15]	Mandibular incisor	Chronic periapical inflammation	Full thickness rectangular flap with healing by secondary intention
Mucosal [16]	Maxillary premolar	Buccally inclined root, chronic periapical inflammation	Laterally positioned flap
Mucosal [17]	Maxillary premolar	Chronic periapical inflammation	Connective tissue graft
